# Candiduria in Hospitalized Patients and Identification of Isolated Candida Species by Morphological and Molecular Methods in Ilam, Iran

**Published:** 2019-01

**Authors:** Afagh FAZELI, Parivash KORDBACHEH, Ali NAZARI, Roshanak DAIE GHAZVINI, Hossein MIRHENDI, Mahin SAFARA, Heidar BAKHSHI, Razieh YAGHOUBI

**Affiliations:** 1. Department of Parasitology and Medical Mycology, School of Public Health, Tehran University of Medical Sciences, Tehran, Iran; 2. Department of Infectious Diseases, School of Medicine, Ilam University of Medical Sciences, Ilam, Iran

**Keywords:** Hospitalized patients, Candiduria, *Candida albicans*, *Candida* spp., Yeast

## Abstract

**Background:**

Candiduria in hospitalized patients may represent contamination, colonization, or urinary tract infections. On the other hand, candidemia and upper urinary tract infection could be the complications of candiduria. The aim of this study was to determine candiduria in hospitalized patients and identify isolated *Candida* species by conventional and molecular methods.

**Methods:**

This cross-sectional study was conducted on hospitalized patients in Imam Khomeini and Mostafa Khomeini hospitals in Ilam, western Iran from Jan to Dec 2016. Urine samples of hospitalized patients were collected during a period of 4 months for diagnosis of candiduria. Primary identification was done by conventional methods. PCR profile was carried out using phenol-chloroform method and confirmed using restriction fragment length polymorphism (PCR-RFLP) technique by *MspI* restriction enzyme.

**Results:**

Candiduria was diagnosed in 18 (9.2%) cases from a total of 195 patients. Isolated yeasts were identified as *C. albicans* (n: 13), *C. glabrata* (n: 5), and *C. parapsilosis* (n: 1) in the one case both *C. albicans* and *C. glabrata* were isolated from a urine sample.

**Conclusion:**

*Candida* urinary tract infection is becoming increasingly common in hospitalized patients but, differentiation fungal colonization from infection and identification of etiologic agents for optimal treatment is necessary.

## Introduction

Since the hospitalized patients are usually associated with treatment by broad-spectrum antimicrobial agents, corticosteroids, immunosuppressive and cytotoxic drugs, urinary tract fungal infection is remarkably increased in them. On the other hand, there are many other risk factors among the patients which are including old age, diabetes mellitus, chronic renal failure, dialysis, renal transplantation, and malformation of urinary tract (1). The presence of *Candida* in urine may be due to colonization of the yeast in perineum or urinary catheter or may be related to invasive infections such as pyelonephritis/cystitis or disseminated candidiasis (2). In healthy people *Candida* species are unusual reason for urinary tract infections (UTIs) but, this fungal infection is more common in hospitalized patients. The urinary tract may be invaded either by bloodstream or retrograde via the urethra and bladder. *Candida* species virulence factors such as phenotypic switching, dimorphism, and hydrolytic enzymes have essential role in colonization and invasion of urinary tract system. Therefore, the presence of yeast in the urine could be indicative of invasive urinary tract infection especially in critically ill patients (3).

In spite of several studies, the true incidence of candiduria and response to treatment is not well defined. The resistance of *Candida* spp. especially non-*albicans Candida* (NAC) spp. to antifungal drugs also should be considered. Although *C. albicans* is the most common (50%–65%) etiologic agent of candiduria, the prevalence of infections caused by NAC spp. has increased in recent years (4, 5).

The aim of this study was to determine the frequency of candiduria in hospitalized patients and identify isolated *candida* species by conventional and molecular methods in order to early diagnosis of invasive fungal infections in these patients.

## Materials and Methods

This cross-sectional study was conducted on hospitalized patients in Imam Khomeini and Mostafa Khomeini hospitals in Ilam, western Iran from Jan to Dec 2016.

Informed consent to participate in this study was signed by patients, after that the study was approved by the Ethics Committee of Tehran University of Medical Sciences.

Urine samples were obtained in sterile urine bottles. Totally, 10 μl of the urine sample was cultured on CHROM agar candida medium (CHROM agar candida, France) before and after centrifugation. The culture media were incubated at 35 °C for 48 h and evaluated based on color and number of growth colonies. If no growth was seen the media were incubated for several more days. Isolated colonies also were cultured on corn meal agar medium (Micromedia, Hungary) supplemented with Tween 80 for identification of *Candida* species based on their morphology. In this study urine, wet-mount examination was done to detect fungal elements in the urine sediment.

PCR-RFLP method was performed for definite identification of species. All isolated strains subcultured on Sabouraud Dextrose Agar medium (Sigma, USA) and genomic DNA were extracted using the phenol-chloroform method. PCR amplification was performed using the universal primers ITS1 (forward: 5′ -TCC- GTA- GGT- GAA -CCT –GCG- G-3′) and ITS4 (reverse: 5′ TCC- TCC- GCT- TAT- TGA -TAT -GC-3′). PCR products were digested by *MspI* restriction enzyme and restriction fragments were separated by 2% agarose gel electrophoresis (6).

Demographic data were analyzed using Chi-Square test by SPSS version 22 (Chicago, IL, USA) ver. 15 and a *P*<0.05 was considered statistically significant.

## Results

**Fig. 1:**
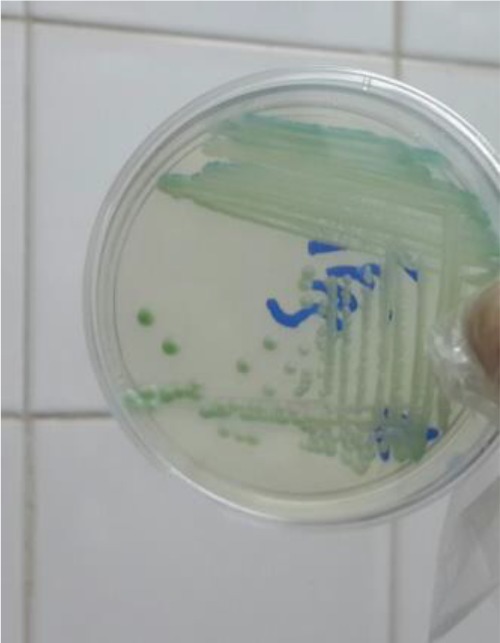
*Candida albicans* isolated from urine culture on CHROMagar candida incubated at 35 °C for 48 h

**Fig. 2:**
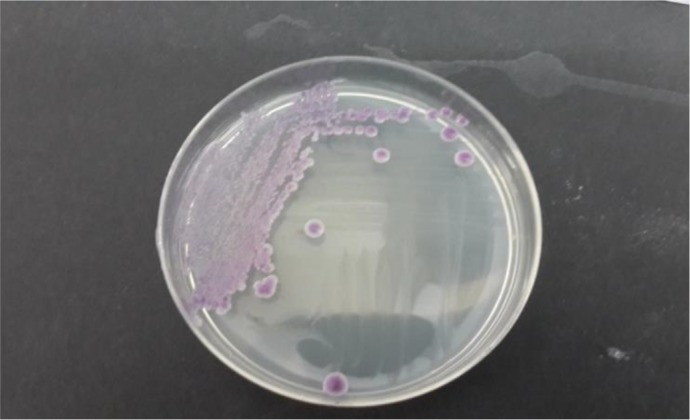
*Candida glabrata* isolated from urine culture on CHROM agar candida incubated at 35 °C for 48

**Fig. 3:**
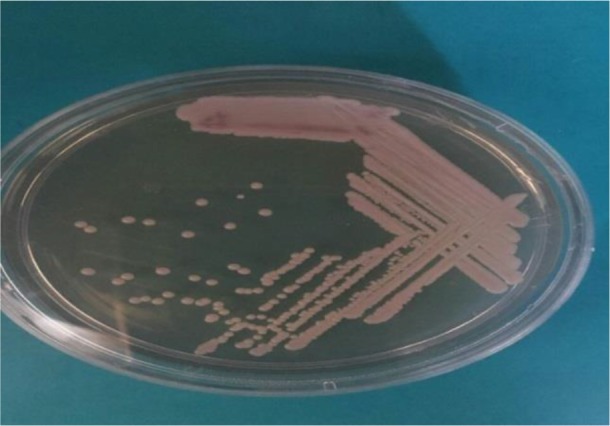
*Candida parapsilosis* isolated from urine culture onCHROM agar candida incubated at 35 °C for 48

**Fig. 4:**
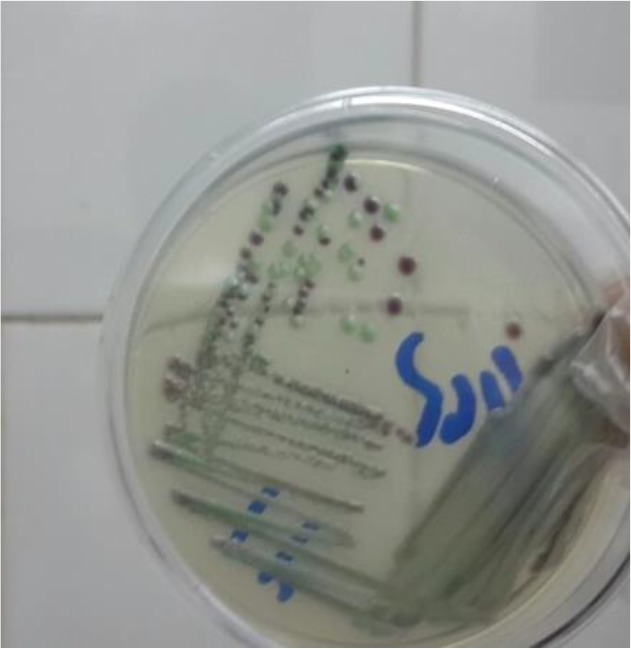
*Candida glabrata and C. albicans* isolated together from urine culture on CHROM agar candida incubated at 35 °C for 48

**Fig. 5:**
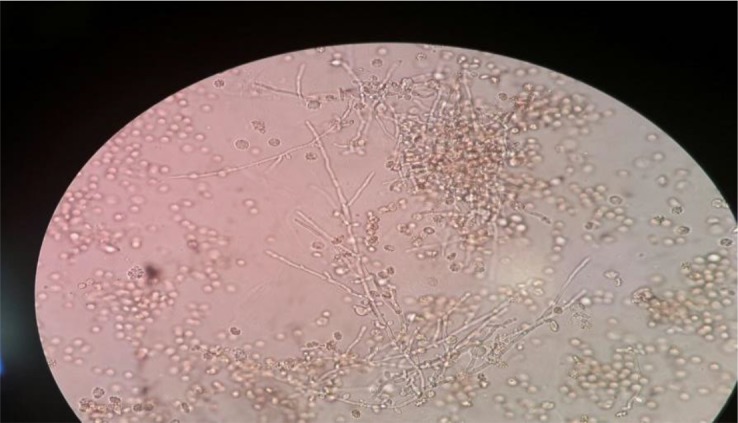
Budding yeast cells, pseudohyphae and true mycelium in direct examination of urine sediment (×400)

**Fig. 6:**
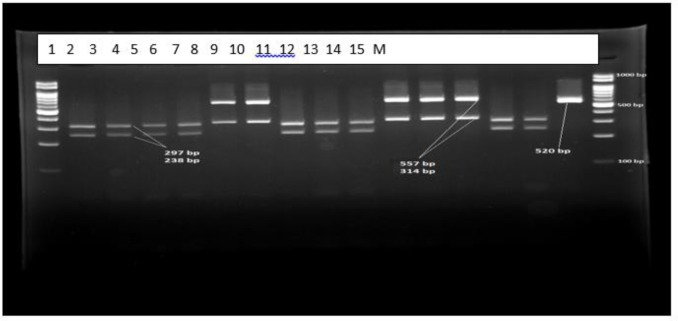
Patterns of PCR-RFLP products of *Candida* spp. isolated from candiduria after digestion by the restriction enzyme *MspI*. Lanes of 1,2,3,4,7,8,9,13,14 represent *C. albicans* (238, 297 bp); 5,6,10,11,12 *C. glabrata* (557, 314 bp) and 15 *C. parapsilosis* (520 bp). Lane M is 100 bp ladder molecular size marker

Overall, 195 patients who included 80 (41.02%) males and 115 (58.97%) females aged 14–93 yr (median age 53 yr) were enrolled. None of the patients received antifungal drugs for prophylaxis or treatment. Underlying conditions in understudy patients were include: diabetes mellitus, antibiotic therapy, indwelling urinary catheters and blood pressure. The urine cultures of 18 (9.7%) patients were yielded *Candida* species and colony count was different from 2000 to >10^5^ cfu/ml (Table 1). By morphological methods, some of isolates were identified as pure, including *C. albicans* with light green colonies (n: 13), (Fig. 1), *C. glabrata* with purple to pink colonies (n: 5), (Fig. 2) and *C. parapsilosis* with pink-cream colonies (n: 1), (Fig. 3). In urine culture of a 28 yr old female hospitalized in ICU, both colonies of *C. albicans* and *C. glabrata* were growth as mixed (Fig. 4). Direct examination showed fungal elements in the sediment of urine samples in 17 patients with candiduria and budding yeast cells, pseudohyphae, and true mycelium were detected (Figs. 5, 6).

**Table 1:** The comparison of morphological and molecular methods in diagnosis of candiduria in hospitalized patients in Imam Khomeini and Mostafa Khomeini hospitals in Ilam, western Iran

***NO.***	***Direct Smear***	***Colony count in urine(before centrifugation)***	***Colony count in 1ml urine sediment(after centrifugation)***	***Culture on CHOROM agar CANDIDA***	***PCR-RFLP***
1	Budding yeast cells, pseudo hyphae			* C. albicans * with green light colonies	* C. albicans *
2	Budding yeast cells, pseudo hyphae	8000 >10 ^ 5 ^	10000 >10 ^ 5 ^	* ” *	* C. albicans *
3	Budding yeast cells, pseudo hyphae and true mycelium	10000	130000	* ” *	* C. albicans *
4	Budding yeast cells, pseudo hyphae	7000	12000	* ” *	* C. albicans *
5	Budding yeast cells, pseudo hyphae			* C. albicans * with green light & * C.glabrata * with purple to pink colonies	* C. albicans * and * C.glabrata *
6	_	>10 ^ 5 ^ 1000	>10 ^ 5 ^ 2000	* C. albicans * with green light colonies	* C. albicans *
7	Budding yeast cells, pseudo hyphae, true mycelium	8000	10000	* ” *	* C. albicans *
8	Budding yeast cells	>10 ^ 5 ^	>10 ^ 5 ^	* ” *	* C. albicans *
9	Budding yeast cells	3000	5000	* ” *	* C. albicans *
10	Budding yeast cells	>10 ^ 5 ^	>10 ^ 5 ^	* ” *	* C. albicans *
11	Budding yeast cells	5000	7000	* C.glabrata * with purple to pink colonies	* C.glabrata *
12	Budding yeast cells	6000	6000	* ” *	* C.glabrata *
13	Budding yeast cells	4000	10000	* ” *	* C.glabrata *
14	Budding yeast cells, pseudo hyphae	>10 ^ 5 ^	>10 ^ 5 ^	* C. albicans * with green light colonies	* C. albicans *
15	Budding yeast cells, pseudo hyphae	5000	8000	* ” *	* C. albicans *
16	Budding yeast cells, pseudo hyphae	5000	5000	* C. albicans * with green light colonies	* C. albicans *
17	Budding yeast cells, pseudo hyphae	>10 ^ 5 ^	>10 ^ 5 ^	* C.parapsilosis * with pink-cream colonies	* C.parapsilosis *
18	Budding yeast cells	4000	5000	* C.glabrata * with purple to pink colonies	* C.glabrata *

Using PCR-RFLP method, 19 isolated yeasts were identified as *C. cabicans* (n: 13), *C. glabrata* (n: 5), and *C. parapsilosis* (n: 1), respectively (Fig. 6). Molecular findings confirmed the result of morphological method (Table 1). *C. albicans* was the most common *Candida* species (68.42%) isolated from the urine samples followed by *C. glabrata* (26.31%), and *C. parapsilosis* (5.26%).

Overall, 18 cases of candiduria were identified in 7 (8.75%) male and 11(9.6%) female. However, there was not statistically correlation between sex and candiduria (*P*<0.42). None of the women had vulvovaginal candidiasis. In this study significant correlation between diabetes mellitus, antibiotic therapy and indwelling urinary catheters with candiduria was observed (*P*<0.05).

## Discussion

The frequency of UTIs caused by *Candida* spp. has been increased especially in hospitalized patients. Individuals with UTI and underlying diseases should be treated with appropriate antifungal drugs. However, there is no suitable protocol for management of candiduria and differentiation of colonization from UTI is usually difficult. Therefore, treatment of candiduria depends on the patient’s clinical condition (6–8).

In present study, candiduria was observed in 9.2% of patients hospitalized in different wards of two teaching hospitals in Ilam. This rate is compatible with some other studies (9–13). Besides, different from low rate of candiduria was reported in hospitalized patients in Hashemi Nejad hospital in Tehran (14). It may be related to different population of patients and differences in hospital setting. Although candiduria is more common in women and usually associated with vaginal candidiasis (15, 16), in our study none of patients had vaginal *Candida* infection and there was no significant correlation between gender and candiduria. Absence of vaginal candidiasis could be an important factor in this situation. Candiduria is usually associated with diabetes mellitus, indwelling urinary catheter and treatment with antibacterial drugs (17–19). Our study also showed significant correlation (*P*<0.05) between candiduria with above mentioned predisposing factors and emphasizes special attention for *Candida* UTIs in these high-risk patients.

Because of relatively large amount of lipids in cell wall of some *Candida* species, yeast cells may be float in urine (20) thus urine samples were cultured before and after centrifugation but, numbers of colonies yielded from urine sediments were much greater than those yielded from whole urine specimens. This finding implies to importance of using urine sediment to isolate *Candida* species from urine samples.

In the present study, *C. albicans* was the most common (68.42%) isolated species from urine samples followed by *C. glabrata* (26.31%) and *C. parapsilosis* (5.26%)*.* This result is in agreement with the most conducted studies in Iran (9, 10, 14). However, in contrast with some others that showed NAC species was more commonly isolated from the urine samples (11, 21). This could be related to different populations of patients, and also in different geographic regions. In the present study, we use mycological and molecular (PCR-RFLP) methods to identify *Candida* species and in both, the same results were obtained. This finding implies to importance of conventional method in diagnosis of fungal diseases. In direct examination of urine sediments budding yeast cell, pseudohyphae and true mycelium were seen in 17 patients with candiduria. This finding implies to importance and necessity of direct urine examination in diagnosis of candiduria. There is not standard colony counting for differentiation of UTI from urine contamination and in our study colony count was different from 4000 to >10^5^ CFU/ml in positive culture from urine samples. However, these findings need more evaluation and follow up patients especially those with serious predisposing factors and long hospital stay.

## Conclusion

Candiduria in hospitalized patients may represent urinary tract infection and requires early diagnosis and treatment. It is difficult to differentiate urinary infection from colonization and treatment should be considered in related to patient’s condition. In present study, *C*. *albicans* was the most common isolated species but drug-resistant NAC species also should be considered. Obviously, definitive diagnosis of *Candida* UTI needs using new laboratory and molecular methods.

## Ethical considerations

Ethical issues (Including plagiarism, informed consent, misconduct, data fabrication and/or falsification, double publication and/or submission, redundancy, etc.) have been completely observed by the authors.
